# Supplementary material for: Patterns of Intron Gain and Loss in Fungi
Source: PLoS Biol. 2004 Nov 30;2(12):e422. doi: 10.1371/journal.pbio.0020422 (PMC532390; doi:10.1371/journal.pbio.0020422)
Supplement: Table S1 — Also available at http://genes.mit.edu/NielsenEtAl/. (4.3 MB ZIP). [file pbio.0020422.st001.zip › NielsenEtAl/html/1293.html]

AN6653.1.NCU10007.1.MG02813.1.FG08700.1


```
 CLUSTAL W (1.82) Multiple Sequence Alignments - Introns Inserted


Sequence 1: NCU10007.1	542 aa
Sequence 2: FG08700.1	542 aa
Sequence 3: MG02813.1	543 aa
Sequence 4: AN6653.1	528 aa
Alignment Length: 543 aa
Number Identitical Residues: 392 aa
Alignment Score (without introns) 17329


MG02813.1 	MASTESILQGVNVLGTVNESQRKILTPPALAFLALLHRSFNERRKQLLERRKLRQAEIDK
NCU10007.1	MASVETLLQGVTISGPIEEHQRKILTPQALSFVALLHRSFNQTRKNLLERRQLRQAEIDR
FG08700.1 	MASLDSVLQGVSVSGKVDEIHRKILTPEALGFLALLHRSFNERRKNLLERRKARQAELDR
AN6653.1  	MSQVDAQLKDVAILGSVSNEARKILTKEACAFLAILHRTFNPTRKALLQRRVDRQAEIDK
          	*:. :: *:.* : * :.:  *****  * .*:*:***:**  ** **:**  ****:*:

MG02813.1 	GVLPDFLPETRHIRENSTWKGAAPAPGLVDRRVEITGPTDRKMVVNALNSNV~WTYMADF
NCU10007.1	GVLPDFLPETKHIRENPTWKGAPPAPGLVDRRVEITGPTDRKMVVNALNSDV~YTYMADF
FG08700.1 	GVLPDFLPETKHIRENPTWKGAAPAPGLVDRRVEITGPTDRKMVVNALNANV~YTYMADF
AN6653.1  	GHLPDFLPETKHIRDDPSWKGAPPAPGLVDRRVEITGPTDRKMVVNALNSDV2-------
          	* ********:***::.:****.**************************::*        

MG02813.1 	EA1D~SSAPTWENMINGQVNLYDAVRRQVDFKQGSKEYKLRTDRTLPTLIVRPRGWHLEE
NCU10007.1	E-~D~SSAPTWANMVNGQVNLYDAIRRQIDFKQGPKEYKLRTDRTLPTLIVRPRGWHLEE
FG08700.1 	E-~D1SSAPTWDNMINGQVNLYDANRRQVDFSQGPKEYKLRTDRKLPTLIVRPRGWHLEE
AN6653.1  	--~-~--APTWDNMINGQINLYDAIRRQVDFKQGQKEYKLRTDRTLPTLIARARGWHLDE
          	       **** **:***:***** ***:**.** *********.*****.*.*****:*

MG02813.1 	KHVTIDGEPISGSLFDFGLYFFHSAHEAVKRGFGPYFYLPKMESHLEARLWNDAFNLGQD
NCU10007.1	KHVTIDGEPVSGSLFDFGLYFFHNAKELVQRGFGPYFYLPKMESHLEARLWNDAFNLAQD
FG08700.1 	RHVTVDGEPISGSLFDFGLYFFHNAFETQRQGFGPYFYLPKMESHLEARLWNDAFNLAQD
AN6653.1  	KHFTVDGEPISGSLFDFGLYFFHNAKELVARGFGPYFYLPKMESHLEARLWNDVFNLAQD
          	:*.*:****:*************.* *   :**********************.***.**

MG02813.1 	YIGMPRGTIRGTVLIETILAAFEMDE~IIYELRDHSSGLNCGRWDYIFSTIKKFRQHPNF
NCU10007.1	YVGIPRGTIRGTVLIETILAAFEMDE~IIYELREHSSGLNCGRWDYIFSTIKKFRNHSSF
FG08700.1 	YIGMPRGTIRGTVLIETILAAFEMDE~IIYELRDHSSGLNCGRWDYIFSVIKKFRNNPNF
AN6653.1  	YIGMPRGTIRGTVLIETITAAFEMEE0IIYELRDHSSGLNCGRWDYIFSFIKKFRQHPNF
          	*:*:************** *****:* ******:*************** *****::..*

MG02813.1 	VLPDRSAVTMTVPFMDAYVKLLIQTCHKRGVHAM~GGMAAQIPIKDDKEANDRAMDGVRA
NCU10007.1	VLPDRSCVTMTVPFMDAYVKLLIQTCHKRGVHAM~GGMAAQIPIKDDKAANDKAMEGVRA
FG08700.1 	VLPDRSAVTMTVPFMESYVKLLIQTCHKRGVHAM~GGMAAQIPIKNDDAANDKAMEGVRA
AN6653.1  	VLPDRSDVTMTVPFMDAYVKLLIKTCHKRGVHAM0GGMAAQIPIKDNAEANDKAMEGVRA
          	****** ********::******:********** **********::  ***:**:****

MG02813.1 	DKLREVRAGHDGTWVAHPALAAIASDIFNKHMPTPNQLFVRREDVHITAMDLLNMNVPGK
NCU10007.1	DKLREARAGHDGTWVAHPALASIALEVFNKHMPTPNQLFNRREDVKIGQQDLLNMNVPGS
FG08700.1 	DKLREVRAGHDGTWVAHPALASIATEIFDKHMPTPNQLFVRRDDVTIGQNDLLNMNVPGT
AN6653.1  	DKLREVRAGHDGTWVAHPALASIASEVFNKYMPTPNQMHVRREDVNITANDLLNTNVPGK
          	*****.***************:** ::*:*:******:. **:** *   **** ****.

MG02813.1 	ITEDGIRKNLYIGLGYMEAWIRGVGCVPINYLM2EDAATAEVSRSQLWQWVKHGVSTAEG
NCU10007.1	ITEEGIRKNLNIGLGYMEAWIRGVGCVPINYLM2EDAATAEVSRSQLWQWVKHGVTTAEG
FG08700.1 	ITEEGIKKNLNIGLGYMEAWIRGVGCVPINYLM~EDAATAEVSRSQLWQWVKHGVSTADG
AN6653.1  	ITEDGIRKNLNIGLSYMEGWLRGVGCIPINYLM0EDAATAEVSRSQLWQWARHGVTTSEG
          	***:**:*** ***.***.*:*****:****** ****************.:***:*::*

MG02813.1 	KKVDKSYALRLLREEADKMAKSLPAGNKMQLASQYFATQVTGEDYADFLTT2LLYNEITT
NCU10007.1	KRVDKSYALKLLKEQTDELASKAPQGNKFNLAAQYFATQVTGEDYADFLTS~LLYNEITS
FG08700.1 	KRVDKSYALKLLKETADQLAASGPKGNKYHLAAQYFSGQVTGEDYADFLTS2LLYNEITQ
AN6653.1  	KKVDKAYALRLLKEQADALAAKGPKGNKFQLAGRYFAGQVTGEDYADFLTS~LLYNEISS
          	*:***:***:**:* :* :* . * *** :**.:**: ************: ******: 

MG02813.1 	PGSARPASKL
NCU10007.1	AGNSLPASKL
FG08700.1 	AGPPSPASKL
AN6653.1  	PG---TASKL
          	.*   .****
```
